# Dehydroepiandrosterone Sulfate (DHEAS) Is an Endogenous Kv7 Channel Modulator That Reduces Kv7/M-Current Suppression and Inflammatory Pain

**DOI:** 10.1523/JNEUROSCI.2307-22.2023

**Published:** 2023-10-25

**Authors:** Lamees Alhassen, Wedad Alhassen, Cindy Wong, Yuxuan Sun, Zelin Xia, Olivier Civelli, Naoto Hoshi

**Affiliations:** ^1^Department of Pharmaceutical Sciences, University of California–Irvine, Irvine, California 92697; ^2^Department of Physiology and Biophysics, University of California–Irvine, Irvine, California 92697

**Keywords:** analgesic, Kv7, pain, steroid hormone, voltage-gated potassium channels

## Abstract

Neuronal Kv7 voltage-gated potassium channels generate the M-current and regulate neuronal excitability. Here, we report that dehydroepiandrosterone sulfate (DHEAS) is an endogenous Kv7 channel modulator that attenuates Gq-coupled receptor-induced M-current suppression. DHEAS reduced muscarinic agonist-induced Kv7-current suppression of Kv7.1, Kv7.2, Kv7.4, or Kv7.5 homomeric currents and endogenous M-currents in rat sympathetic ganglion neurons. However, DHEAS per se did not alter the voltage dependence of these Kv7 homomeric channels or the m1 receptor-induced activation of phospholipase C or protein kinase C. DHEAS-treated Kv7.2 homomeric currents became resistant to depletion of phosphatidylinositol 4,5-bisphosphate (PIP2) induced by voltage-activated phosphatase, Ci-VSP or eVSP. Our computational models predicted a novel binding site for DHEAS in the cytoplasmic domain of Kv7 subunits. A single-point mutation of the predicted key histidine into cysteine in the rat Kv7.2 subunit, rKv7.2(H558C), resulted in a loss of effects of DHEAS on muscarinic Kv7 current suppression. Furthermore, *in vivo* administration of DHEAS in mice of both sexes reduced late phase pain responses in the formalin paw test. However, it did not have effects on early phase responses in the formalin paw test or responses in the hot plate test. Coadministration of a selective Kv7 inhibitor, XE991, and DHEAS eliminated analgesic effects of DHEAS in late phase responses in the formalin paw test. Collectively, these results suggest that DHEAS attenuates M-current suppression by stabilizing PIP2–Kv7 subunit interaction and can mitigate inflammatory pain.

**SIGNIFICANCE STATEMENT** M-current suppression induced by stimulation of Gq-coupled receptors is a form of Kv7 current modulation that can reversibly increase neuronal excitability. This study demonstrates that DHEAS, an endogenous steroid hormone, is a novel Kv7 channel modulator that can attenuate M-current suppression without affecting basal Kv7 channel kinetics. Administration of DHEAS *in vivo* alleviated inflammatory pain in rodents. These results suggest that the degree of M-current suppression can be dynamically regulated by small molecules. Therefore, this novel form of Kv7 channel regulation holds promising potential as a therapeutic target for sensitized nervous activities, such as inflammatory pain.

## Introduction

The *Kcnq* gene family encodes five subtypes of low voltage-activated Kv7 potassium channels, which are known for their activation near the resting membrane potential and minimal inactivation (Jentsch, 2000; Brown and Passmore, 2009). Persistent activity of Kv7 channels at or near the resting membrane potential antagonizes depolarizing stimuli and counteracts the firing of action potentials. Conversely, suppression of neuronal Kv7 channel activity through Gq-coupled receptor activation promotes action potential firing (Brown and Passmore, 2009; Greene and Hoshi, 2017). This Gq receptor-induced Kv7 current suppression has been known as M-current suppression and is most noticeable in neurons (Brown and Passmore, 2009; Greene and Hoshi, 2017). PIP2 is the substrate of phospholipase C(PLC) but is also an essential cofactor for Kv7 channels for voltage sensing and maintaining the ion-conducting conformation (Zhang et al., 2003; Winks et al., 2005; Sun and MacKinnon, 2020; Zheng et al., 2022). Three pathways have been identified for mediating M-current suppression and are closely related to the Gq-coupled receptor pathway. (1) Protein kinase C (PKC) pathway: A selective pool of PKC is anchored to the Kv7.2 subunit through a scaffold protein AKAP79/150. When Gq-coupled receptors are activated, Kv7 anchored PKC phosphorylates Kv7.2 subunits, which reduces the affinity of Kv7.2 toward phosphatidylinositol 4,5-bisphosphate (PIP2) and results in a loss of PIP2 from Kv7 channels (Hoshi et al., 2003; Kosenko et al., 2012). The Kv7.1 subunit is distinct because it does not bind AKAP79/150 and lacks the critical PKC acceptor site for this pathway (Hoshi et al., 2003; Bal et al., 2010). (2) Calcium-dependent pathway: Calmodulin is an auxiliary subunit of Kv7 channels and is required for channel trafficking as well as calcium sensing. When intracellular calcium increases, calcium-calmodulin changes its binding configuration and reduces affinity of the Kv7 subunit toward PIP2, which results in a loss of PIP2 from Kv7 channels (Kosenko and Hoshi, 2013). (3) PIP2 depletion pathway. Consumption of PIP2 by PLC leads to PIP2 depletion and results in a loss of PIP2 from Kv7 channels. The shared end result of these three pathways for M-current suppression is a loss of PIP2 from Kv7 subunits (Greene and Hoshi, 2017).

Small molecular weight compounds have been developed to facilitate Kv7 channel activity for medical intervention. Retigabine and flupirtine are the first-generation compounds of Kv7 channel activators approved for clinical use as an antiepileptic drug and analgesic (Brown and Passmore, 2009). The binding site of retigabine and flupirtine is located within a transmembrane region sandwiched by S5 and S6 segments of Kv7 subunits and drug binding facilitates channel activation with a hyperpolarizing shift of activation voltages (Tatulian and Brown, 2003; Wuttke et al., 2005; Lange et al., 2009; R. Y. Kim et al., 2015). Furthermore, emerging numbers of lipids and plant extracts, including cannabidiol, have been reported to increase Kv7 currents accompanied by hyperpolarizing shifts of voltage dependency, most of which are assumed to bind the retigabine binding site (Larsson et al., 2020; Redford and Abbott, 2022). Recently, an active metabolite of acetaminophen has been shown to increase Kv7 current amplitudes with a similar biophysical modification (van der Horst et al., 2020). On the other hand, these known Kv7 channel modulators cannot open Kv7 channels lacking PIP2 in the channel complex, such as those under M-current suppression because PIP2 is essential for maintaining the ion-conducting conformation of Kv7 channels (Kay et al., 2015; Zhang et al., 2022). The new type of Kv7 modulation described in this paper demonstrates an alternative Kv7 channel modulation via stabilizing Kv7-PIP2 interaction.

DHEAS is the most abundant form of circulating steroid hormones and was once considered as a hydrophilic storage form of dehydroepiandrosterone (DHEA), and these two steroid derivatives (DHEA/DHEAS) were viewed as precursors of sex steroid hormones (Ebeling and Koivisto, 1994). However, numerous physiological functions have been found to be mediated by DHEA/DHEAS, and they are now considered steroid hormones themselves (Ebeling and Koivisto, 1994; Kroboth et al., 1999; Labrie et al., 2005; Maninger et al., 2009; Prough et al., 2016). In this paper, we describe that DHEAS, but not DHEA, testosterone, or β-estradiol, is a Kv7 modulator with active analgesic effects.

## Materials and Methods

### Reagents and expression plasmids

The CFP-PH construct was obtained from Tobias Meyer (Stanford University) through Addgene. Ci-VSP and eVSP were obtained from Yasushi Okamura (Osaka University). Cyt-CKAR was obtained from Alexandra Newton (University of California–San Diego). Human KCNQ1 and KCNQ5 plasmids were obtained from Geoffrey Abbott (University of California–Irvine). Human KCNQ4 plasmid was obtained from Anastasios Tzingounis (University of Connecticut). Rat KCNQ2 expression plasmid has been described (Hoshi et al., 2003). Rat KCNQ2(H558C) constructs were generated by QuickChange II XL site-directed mutagenesis (Agilent Technologies). All PCR-derived constructs were verified by sequencing. DHEAS was purchased from Cayman Chemical.

### Mice

Mice were either C57BL/6 or homozygous Kv7.2(S559A) knock-in mice in C57BL/6 background. Generation of Kv7.2(S559A) mice has been described (Greene et al., 2018).

### Experimental design and statistical analysis

#### Cell culture and transfection

Chinese hamster ovary (CHO) hm1 cells (Smith and Hoshi, 2011) were grown in α minimum essential medium with 5% FBS and 500 μg/ml G418 sulfate. TransIT-LT1 reagent (Mirus Bio) and expression plasmids were used for transient transfections. Superior cervical ganglion (SCG) neurons were prepared from 14- to 17-d-old rats and cultured for 2-4 d as described (Hoshi et al., 2003). DRG neurons were isolated from adult mice and cultured in the same medium used for SCG neurons (Passmore, 2005).

#### Electrophysiological measurements

Patch-clamp recordings were performed at room temperature on isolated CHO hm1 cells using an Axopatch 200B patch-clamp amplifier (Molecular Devices) as described previously (Greene et al., 2017). Perforated patch clamp is used for M-current recording from rat SCG neurons or from mouse DRG neurons as described (Kay et al., 2015). Series resistance compensation was set to 75%-86%. Signals were sampled at 2 kHz, filtered at 1 kHz, and acquired using pCLAMP software (version 10, Molecular Devices). For Ci-VSP and eVSP experiments, sampling frequency was 500 Hz (Kosenko and Hoshi, 2013). Cells were perfused with a solution containing 144 mm NaCl, 5 mm KCl, 2 mm CaCl_2_, 0.5 mM MgCl_2_, 10 mm glucose, and 10 mm HEPES, pH 7.4. Patch pipettes (4-6 mΩ) were filled with intracellular solution containing 135 mm potassium aspartate, 2 mm MgCl_2_, 1 mm EGTA, 0.1 mm CaCl_2_, 4 mm ATP, 0.1 mm GTP, and 10 mm HEPES, pH 7.2. Cells were held at −70 mV for Kv7.1, Kv7.2, and Kv7.4, or −90 mV for Kv7.5 channel recordings. Kv7 currents were evoked by two-step test pulses to 0 mV followed by −50/−60 mV, with 500 ms duration for each step. Kv7 currents were measured at the end of the 0 mV step for time course experiments. For measuring activation curves, Kv7 tail current at −50/−60 mV was measured and normalized to the largest current. SCG or DRG neurons were held at −30 mV and 500 ms steps to −50 mV were given every 10 s. For Ci-VSP experiments, cells were held at −70 mV and 10 s step depolarizations to 60 mV with 2 min interstep intervals applied. A 100 ms voltage step to –60 mV before the test 60 mV step was applied to calculate the linear leak (Kosenko and Hoshi, 2013). For eVSP experiments, cells were held at −70 mV and 2 s step depolarizations to −20 mV followed by 1 s steps to 100 mV; then 4 s steps to −20 mV were applied before and 2 min after 10 μm DHEAS application.

#### Live cell imaging

All imaging procedures have been previously described (Kosenko et al., 2012; Kosenko and Hoshi, 2013). Transiently transfected CHO hm1 cells were plated onto 18 mm glass coverslips 24 h after transfection. Images were acquired 48 h after transfection with an inverted microscope IX-81 (Olympus) and an ImageEM CCD camera (Hamamatsu Photonics) and processed with MetaMorph 7.6.3 (Molecular Devices). For TIRF experiments, the excitation source was a 445 nm diode laser (Coherent) with an acousto-optical tunable filter. For epi-fluorescent experiments, excitation light was generated by λ LS (Sutter Instrument) and passed through S436/10× or S500/20× filters. Dual-emission images of cyt-CKAR experiments were obtained simultaneously through a dual-view module (Photometrics) with ET535/30m, ET480/40m emission filters and a T505lpxr dichroic mirror (Chroma Technology). For cyt-CKAR, excitation light through the S435/10× filter and signals from CFP (direct CFP) and YFP (indirect YFP) channels were used to calculate ratio of these two channels as an indicator of FRET signals as described previously (Hoshi et al., 2010; Smith and Hoshi, 2011).

#### Computational modeling

Computational binding analyses were performed using Autodock vina (Trott and Olson, 2010) in Pyrx software (pyrx 0.8) (Dallakyan and Olson, 2015). The template structure for the functional Kv7.1 was obtained from the published structure of Kv7.1-KCNE3-CaM-PIP(+) (pdb: 6v01) (Sun and MacKinnon, 2020) with a modification of removal of KCNE3 from the original structure. The template for inactive Kv7.1-PIP(–) structure was obtained from pdb: 6v00 with a modification of removal of KCNE3 (Sun and MacKinnon, 2020). Removal of KCNE3, and virtual mutations for a Kv7.2 template were performed using MacPymol (version 1.5.0.4). Kv7.4-CaM-PIP2 structure has been obtained from pbd; 7vnp (Zheng et al., 2022). 3D molecule models of steroids were obtained from the PubChem website (https://pubchem.ncbi.nlm.nih.gov) or from the Zinc database (https://zinc15.docking.org). Obtained predicted binding configurations were visualized in MacPymol.

### Behavioral tests

Mice were group-housed and maintained on a 12 h light/dark cycle with food and water available *ad libitum*. Mice of both sexes were used. All mice were observed after injections to ensure proper health. Any mouse that seemed to be in distress was excluded from the study. DHEAS was injected into mice intraperitoneally (40 mg/kg, i.p.). The DHEAS injection solution was freshly prepared on the day of experiments in PBS (1× PBS) from a stock solution in DMSO.

#### Hot plate assay

To establish the acute antinociceptive effects of DHEAS, foot withdrawal latency in the hot plate assay was measured at 30 min after injection. Mice were habituated to handling and the hot plate testing apparatus for 3 d before conducting assays. Hot plate was set at 52°C, and mice were tested first with no injection to ensure normal baseline on heat, which ranged between 4 and 8 s. Hotplate assay was performed as previously described (Wang et al., 2016; Jang et al., 2018; Alhassen et al., 2021). The cutoff time for this assay was set at 50 s to avoid any thermal injury to mice.

#### Formalin paw assay

The formalin paw assay was performed as described (Hunskaar and Hole, 1987). Mice were habituated in the testing arena for 10 d before formalin paw test. Vehicle (PBS, i.p.), DHEAS (40 mg/kg, i.p.), and/or XE991 (2 mg/kg, i.p.) was administered 10 min before the formalin injection; 10 µl of 2% formalin solution was administered into the dorsal surface of the right hind paw using a Hamilton syringe with a 30G needle. After formalin injection, mice were placed in the testing arena, and paw licking was observed for the duration of 50 min. The amount of time mice spent licking injected paw for the first 15 min is defined as the early phase, and from 20 to 50 min as the late phase. Paw licking duration was examined and recorded by experimenters blinded to genotypes and drug treatments. All animals subjected to the formalin paw assay were killed immediately after the assessment to alleviate inflammation induced distress.

### Statistics

All pooled values are expressed as mean ± SEM. Mann–Whitney test was used for two comparison groups. Paired *t* test was used for comparing responses before and after treatments in the same cells. For testing more than two comparison groups with different variances among comparison groups, the Kruskal–Wallis test followed by Dunn's multiple comparisons test was used. Two-way ANOVA was used to evaluate effects of treatments and two phases in the formalin paw tests. Statistical significance was defined as two-tailed *p* < 0.05. *p* values for statistical analyses were calculated using Prism 6 (GraphPad Software).

### Study approval

All experiments were conducted according to National Institute of Health guidelines for animal care and use. All protocols used in this study were approved by the Institutional Animal Care and Use Committee of the University of California–Irvine and the Institutional Biosafety Committee of the University of California–Irvine.

## Results

### DHEAS, but not related steroid hormones, attenuated suppression of Kv7 current or the M-current induced by muscarinic stimulation

**Figure 1.**
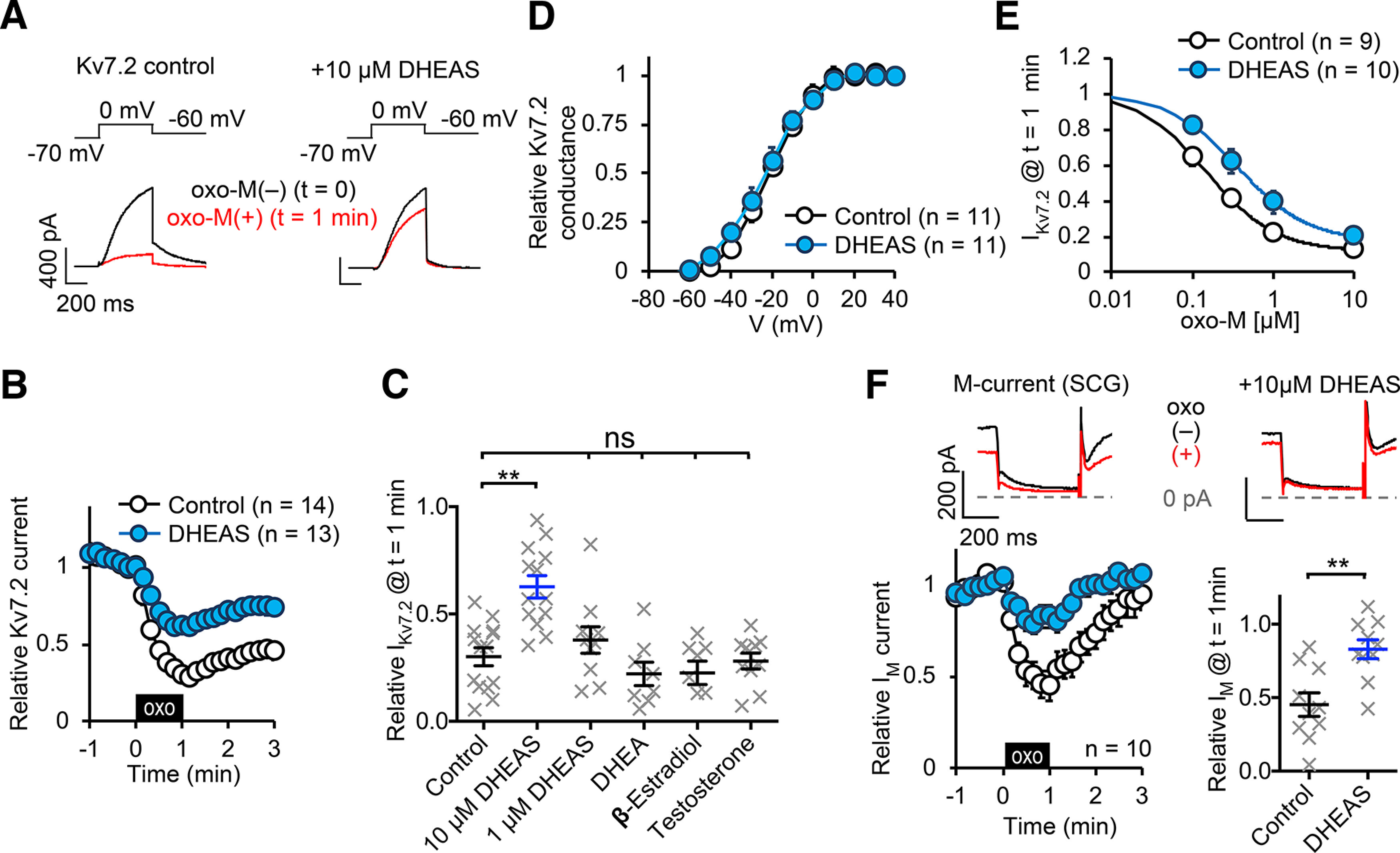
Characterization of effects of DHEAS on Kv7.2 homomeric current and M-current suppression induced by oxo-M. ***A***, Representative Kv7.2 current traces represent before [oxo(–)] and after 1 min application of 0.3 μm oxo-M [oxo(+)]. The voltage protocol is indicated. ***B***, Time course of pooled Kv7.2 current responses to 0.3 μm oxo-M with or without 10 μm DHEAS. Black box represents presence of oxo-M. ***C***, Scatter plots represent relative amplitudes of Kv7.2 current after 1 min application to 0.3 μm oxo-M from individual cells shown in ***B*** and results of indicated steroid hormones tested (all tested with 10 μm, except 1 μm DHEAS). ***D***, Effects of 10 μm DHEAS on Kv7.2 channel activation curves. ***E***, Dose–response relationship of oxo-M to Kv7.2 current with or without 10 μm DHEAS. ***F***, Summary of M-current recording from rat SCG neurons. Top panels, Representative current traces of current suppression by 0.3 μm oxo-M and effects of 10 μm DHEAS. Bottom left, Time course of pooled M-current response to 1 min application of 0.3 μm oxo-M. Bottom right, Scatter plot of individual cells at *t* = 1 min. Data are mean ± SEM. Not significant > *p* = 0.05. ** < *p* = 0.01.

Homomeric Kv7.2 channels were transiently expressed in CHO hm1 cells, a stably expressing clone of human m1 muscarinic acetylcholine receptor in Chinese hamster ovary cells, and Kv7.2 current suppression was studied by activating m1 receptors by 0.3 μm oxotremorine-M (oxo-M) as a model of M-current suppression as we described previously (Kosenko et al., 2012). In control cells, 0.3 μm oxo-M application reduced Kv7.2 current to 0.33 ± 0.04 (*n* = 11) of the reference current amplitude measured before application (Fig. 1*A-C*). After application of DHEAS for 2-4 min before oxo-M application, DHEAS-treated cells showed attenuated oxo-M-induced Kv7.2 current suppression with increased residual Kv7.2 current after oxo-M exposure of 0.61 ± 0.05 (*n* = 13) compared with the reference current amplitude (*p* < 0.01, Kruskal–Wallis test, Fig. 1*A-C*). Two to 4 min treatments with a reduced concentration of 1 μm DHEAS did not show effect on oxo-M-induced Kv7.2 current suppression [0.37 ± 0.06 to the reference (*n* = 10), Fig. 1*C*]. Since DHEAS can be metabolized into other steroid hormones, we tested related steroid hormones with treatments for 2-4 min: 10 μm DHEA, 10 μm testosterone, 10 μm β-estradiol. These steroid hormones did not have any effects on oxo-M-induced Kv7.2 current suppression (Fig. 1*C*). Currently known Kv7 channel modulators, such as retigabine, typically increase currents accompanied by a shift of activation voltages. In contrast, DHEAS application per se did not modify Kv7.2 current amplitudes evoked by 500 ms voltage steps to 0 mV (0.94 ± 0.06 of the control, *p* = 0.17, *n* = 13, paired *t* test). DHEAS also did not alter half-activation voltages of Kv7.2 currents: control Kv7.2, V_1/2_ = −22.5 ± 3.4 mV (*n* = 8); DHEAS-treated Kv7.2 −25.1 ± 3.5 mV (*n* = 8), *p* = 0.27, Mann–Whitney test (Fig. 1*D*). To further characterize the effect of DHEAS, we measured dose–response relationships of oxo-M to Kv7.2 current suppression. A half maximally inhibitory concentration (IC_50_) of oxo-M in control CHO hm1 cells was 0.15 ± 0.03 μm (*n* = 9) and 10 μm DHEAS treatment shifted IC_50_ to 0.50 ± 0.11 μm (*p* = 0.013, Mann–Whitney test, Fig. 1*E*). On the other hand, both conditions reached to a similar maximal Kv7.2 current suppression at 10 μm oxo-M (Fig. 1*E*). We also tested endogenous M-currents in rat SCG neurons, which have been extensively studied for M-current suppression; 10 μm DHEAS attenuated M-current suppression in these neurons (*p* = 0.002, Mann–Whitney test, Fig. 1*F*). These results suggest that DHEAS is an endogenous circulating hormone that can attenuate M-current suppression. We also tested DRG neurons for modulation of M-current suppression by DHEAS. We detected M-currents in most of small DRG neurons tested as previously reported (Passmore et al., 2003). However, these neurons showed low responding rate to 10 μm oxo-M (2 of 16 small neurons) or 100 nm bradykinin (0 of 3 small neurons), which align with previous findings that the dominant muscarinic receptor subtype in DRG is M_2_ and a limited pool of responding small neurons to bradykinin (Tata et al., 2000; Liu et al., 2010). The presence of a significant population of nonresponding neurons precluded evaluation of DHEAS effects in DRG neurons.

**Figure 2.**
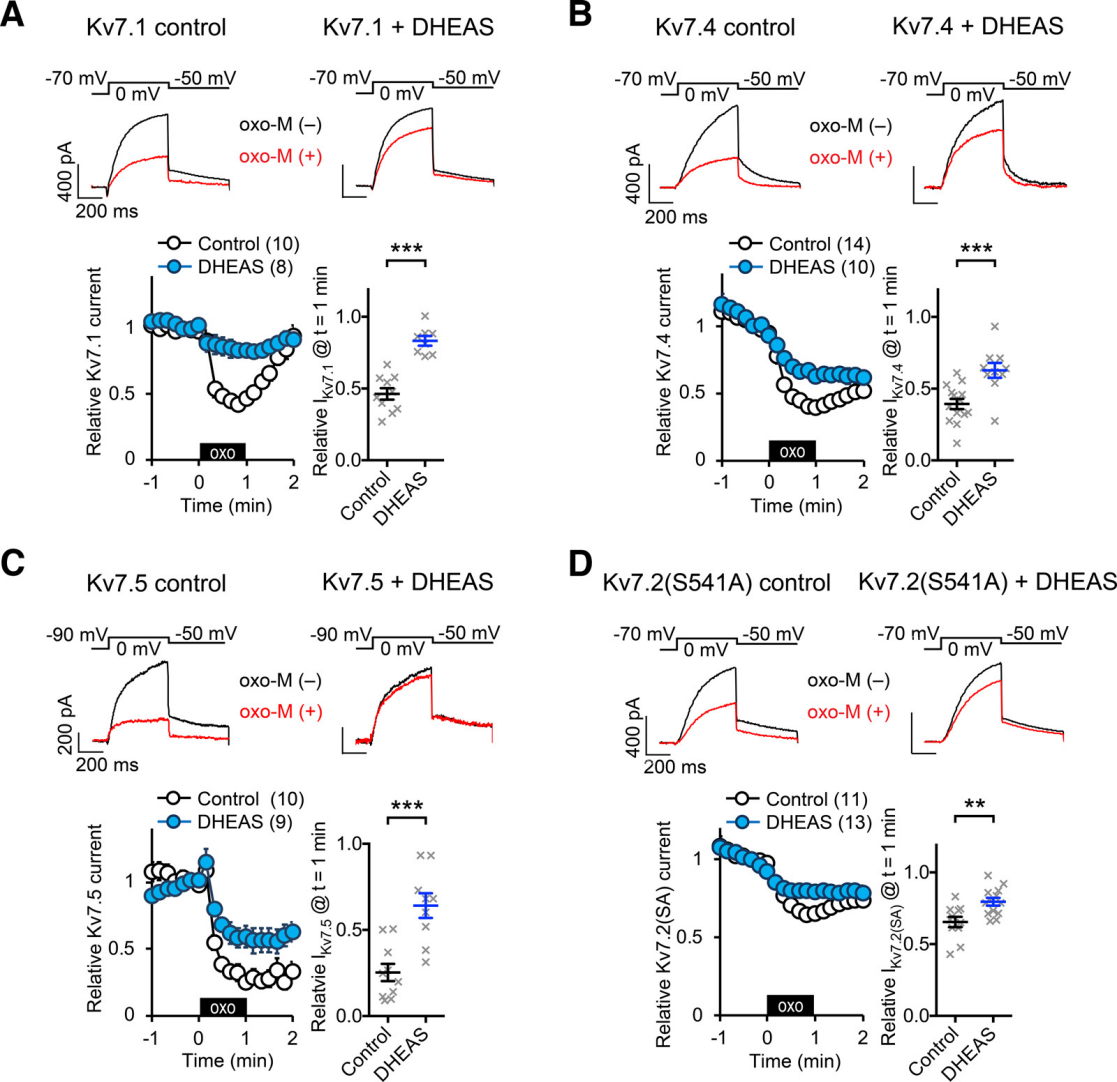
Effects of 10 μm DHEAS on other homomeric Kv7 currents. ***A***, Effects of DHEAS on oxo-M induced suppression of homomeric Kv7.1 current. Top, Representative current traces of Kv7.1 homomeric channels before [oxo-M(–)], and 1 min after [oxo-M(+)] 0.3 μm oxo-M application. Bottom left, Time courses of pooled current responses to 0.3 μm oxo-M application from control cells (open circle) and 10 μm DHEAS-treated cells (blue filled circle). Black box represents presence of oxo-M. Bottom right, Scatter plot represents responses of individual cells at *t* = 1 min. ***B***, Effects of DHEAS on oxo-M induced suppression of homomeric Kv7.4 current. Top, Representative current traces of homomeric Kv7.4 currents before [oxo-M(–)], and 1 min after [oxo-M(+)] 0.3 μm oxo-M application. Bottom left, Time courses of pooled current responses to 0.3 μm oxo-M application from control cells (open circle) and 10 μm DHEAS-treated cells (blue filled circle). Bottom right, Scatter plot represents responses of individual cells at *t* = 1 min. ***C***, Effects of DHEAS on oxo-M induced suppression of homomeric Kv7.5 current. Top, Representative current traces of homomeric Kv7.5 currents before [oxo-M(–)], and 1 min after [oxo-M(+)] 0.3 μm oxo-M application. Bottom left, Time courses of pooled current responses to 0.3 μm oxo-M application from control cells (open circle) and 10 μm DHEAS-treated cells (blue filled circle). Bottom right, Scatter plot represents responses of individual cells at *t* = 1 min. ***D***, Effects of DHEAS on oxo-M induced suppression of homomeric rKv7.2(S541A) currents. Top, Representative current traces of homomeric rKv7.2(S541A) channels before [oxo-M(–)], and 1 min after [oxo-M(+)] 0.3 μm oxo-M application (top). Bottom left, Time courses of pooled current responses to 0.3 μm oxo-M application from control cells (open circle) and 10 μm DHEAS-treated cells (blue filled circle). Bottom right, Scatter plot represents responses of individual cells at *t* = 1 min. Data are mean ± SEM. *** < *p* = 0.001. ** < *p* = 0.01.

Since all subtypes of the Kv7 channel family share highly conserved modular structures (Jentsch, 2000), we investigated whether DHEAS has a similar effect on other Kv7 subtypes. To this end, Kv7.1, Kv7.4, or Kv7.5 homomeric channels were expressed in CHO hm1 cells and effects of 10 μm DHEAS on responses to 0.3 μm oxo-M were examined. In nontreated control cells, Kv7.1 homomeric currents showed less current suppression after 1 min application of 0.3 μm oxo-M compared with that of Kv7.2 currents, which is consistent with the lack of the PKC pathway in this subtype (Hoshi et al., 2003; Greene and Hoshi, 2017). Importantly, 10 μm DHEAS treatment attenuated Kv7.1 current suppression (*p* < 0.0001, Mann–Whitney test, Fig. 2*A*). DHEAS treatment did not change voltage dependence of activation of Kv7.1 channels: control V_1/2_ = −10.1 ± 3.2 mV (*n* = 11), DHEAS V_1/2_ = −11.5 ± 2.7 mV (*n* = 9), *p* = 0.60, Mann–Whitney test. For homomeric Kv7.4 currents, DHEAS attenuated oxo-M induced current suppression (*p* = 0.0009, Mann–Whitney test, Fig. 2*B*) with unaltered activation voltage: control V_1/2_ = −18.4 ± 1.7 mV (*n* = 14), DHEAS V_1/2_ = −17.5 ± 1.3 mV (*n* = 15), *p* =0.70, Mann–Whitney test. For homomeric Kv7.5 currents, oxo-M-induced current suppression was also attenuated by 10 μm DHEAS treatment (*p* = 0.0004, Mann–Whitney test, Fig. 2*C*) without affecting activation voltage: control V_1/2_ = −42.6 ± 2.2 mV (*n* = 14), DHEAS V_1/2_ = −47.7 ± 4.9 mV (*n* = 6), *p* = 0.15, Mann–Whitney test. Because the Kv7.1 subunit lacks the AKAP/PKC-mediated pathway toward M-current suppression (Hoshi et al., 2003; Greene and Hoshi, 2017), we tested rat Kv7.2(S541A) mutant channels, where the PKC-dependent regulation in Kv7.2 channels has been removed, to confirm whether DHEAS can disrupt Kv7 current suppression by non-PKC mechanisms (Hoshi et al., 2003; Greene and Hoshi, 2017). As expected, DHEAS also attenuated oxo-M induced rKv7.2(S541A) current suppression (*p* = 0.0052, Mann–Whitney test, Fig. 2*D*). These results indicate that DHEAS attenuates Gq-receptor-induced Kv7 current suppression regardless of Kv7 subtypes and mediating pathways.

### DHEAS-treated Kv7.2 currents became resistant to PIP2 depletion

**Figure 3.**
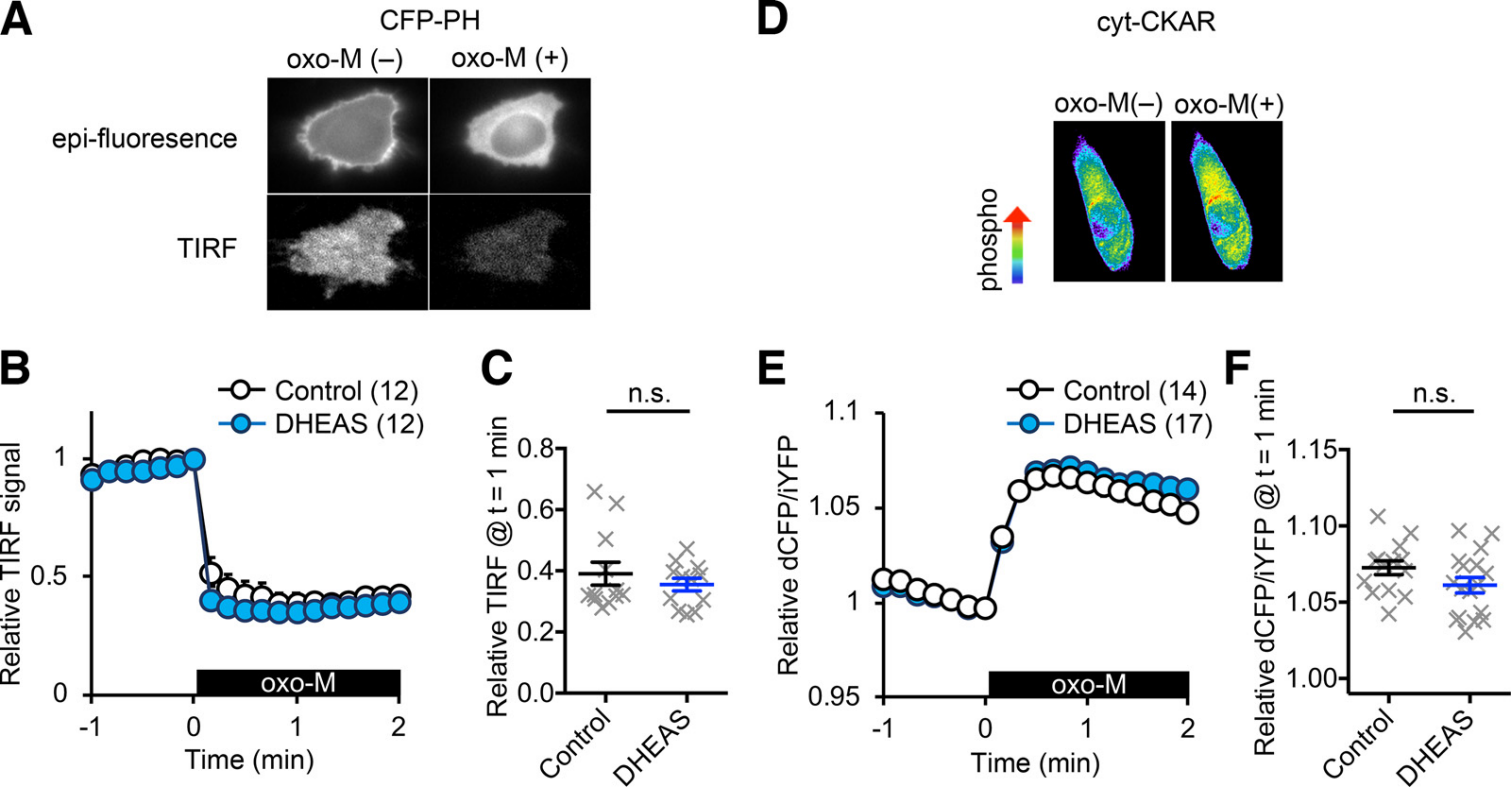
DHEAS effects on PLC and PKC activation. ***A***, Representative epi-fluorescent and TIRF images from the same cell before [oxo-M(–)] and after [oxo-M(+)] application of 0.3 μm oxo-M. ***B***, Time course of pooled relative TIRF signals showing translocation of CHP-PH from the plasma membrane after application of 0.3 μm oxo-M in control cells (open circle) and 10 μm DHEAS-treated cells (blue filled circle). Black box represents presence of oxo-M. ***C***, Scatter plot at *t* = 1 min from ***B*** showing responses of individual cells. ***D***, Representative pseudo-color images of cyt-CKAR responses to 0.3 μm oxo-M. ***E***, Time course of pooled cyt-CKAR responses to 0.3 μm oxo-M. ***F***, Scatter plot at *t* = 1 min from ***E*** showing responses of individual cells. Data are mean ± SEM. Not significant > *p* = 0.05.

Next, we analyzed the mechanism of how DHEAS attenuates Kv7 current suppression. It has been reported that DHEAS can directly modulate G-coupled receptor signaling (Perez-Neri et al., 2008). To eliminate this possibility, we measured oxo-M-induced activation of PLC and PKC in CHO hm1 cells using live-cell imaging. The PH domain of PLCδ fused with CFP (CFP-PH) is a fluorescent probe that binds to PIP2 and IP3 (Hirose et al., 1999; Zhang et al., 2003). Therefore, activation of PLC induces translocation of CFP-PH from the plasma membrane to the cytoplasm. Membrane localization of CFP-PH was measured by TIRF (total internal reflection fluorescent) signal as described (Winks et al., 2005; Kosenko and Hoshi, 2013). Application of 0.3 μm Oxo-M induced reduction of TIRF signals (Fig. 3*A*). DHEAS-pretreated cells showed equivalent reduction of TIRF signals that were triggered by oxo-M, suggesting no alternation in PLC activation (*p* = 0.67, Mann–Whitney test, Fig. 3*A*). Cytosolic CKAR is a FRET reporter for PKC activity at the cytoplasm (Gallegos et al., 2006; Hoshi et al., 2010). Control and DHEAS-treated cells showed equivalent PKC activity after application of 0.3 μm oxo-M (*p* = 0.1, Mann–Whitney test, Fig. 3*B*). These results suggest that DHEAS does not alter the global m1 muscarinic receptor signaling.

**Figure 4.**
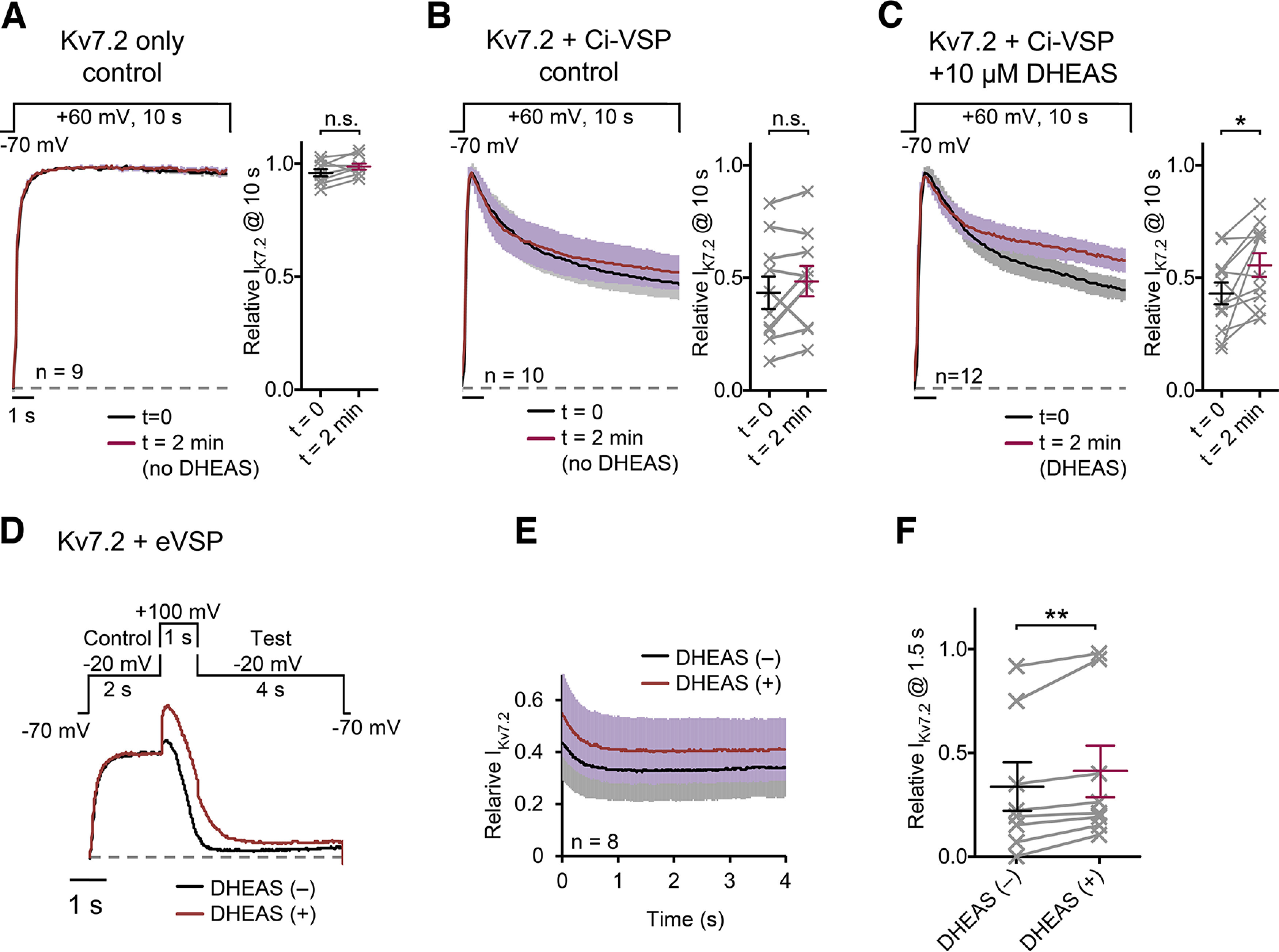
Effects of DHEAS on Kv7.2 current during Ci-VSP-mediated PIP2 depletion. ***A***, Pooled normalized current traces from cells expressing only Kv7.2 channel during 10 s voltage steps to 60 mV. Currents were normalized to the peak current during the depolarizing steps and measured with a 2 min interval (*t* = 0, and 2 min). Right, Pairs of responses from individual cells at the end of 10 s steps at *t* = 0 and *t* = 2 min. ***B***, Pooled normalized current traces from cells expressing Kv7.2 and Ci-VSP (Kv7.2 + Ci-VSP) during 10 s voltage steps to 60 mV. Currents were normalized to the peak current during the depolarizing step and measured with 2 min interval (*t* = 0, and 2 min). Right, Pairs of responses from individual cells at the end of 10 s steps at *t* = 0 and *t* = 2 min. ***C***, Pooled normalized current traces from cells expressing Kv7.2 and Ci-VSP (Kv7.2 + Ci-VSP) before (*t* = 0) and after 2 min application of 10 μm DHEAS (*t* = 2 min). Right, Pairs of responses of individual cells at the end of 10 s steps from before (*t* = 0) and after DHEAS application (*t* = 2 min). ***D***, Representative normalized current traces from a cell expressing Kv7.2 and eVSP (Kv7.2 + eVSP) before and after 2 min application of 10 μm DHEAS. Voltage protocol (control −20 mV, 2 s; PIP2 depletion 100 mV, 1 s; test −20 mV, 4 s) is shown. ***E***, Pooled normalized current traces at the test step. ***F***, Paired responses of individual cells at 1.5 s shown in ***E*** before and after 2 min application of DHEAS. Data are mean ± SEM. Not significant > *p* = 0.05. * < *p* = 0.05. ** < *p* = 0.01.

Because muscarinic suppression of Kv7.2 current is mediated by a loss of PIP2 from Kv7.2 subunits, another potential mechanism for attenuation of Kv7 current suppression is the capacity of DHEAS to sustain the ion-conducting Kv7-PIP2 conformation even at conditions with decreased PIP2 levels. To test this idea, we examined how DHEAS modifies PIP2 sensitivity of Kv7.2 homomeric current using a voltage-activated phosphatase, Ci-VSP, which catalyzes PIP2. PIP2 depletion was induced by coexpression of Ci-VSP with Kv7.2 and 10 s depolarization steps to 60 mV (Murata and Okamura, 2007; Kosenko and Hoshi, 2013; Kang et al., 2014). Without Ci-VSP, Kv7.2 currents themselves did not show apparent current inactivation or decrease during 10 s depolarization steps to 60 mV (Fig. 4*A*). Kv7.2 currents coexpressed with Ci-VSP (Kv7.2 + Ci-VSP) showed gradual current decay during 10 s depolarization steps (Fig. 4*B*). The degree of Kv7.2 + Ci-VSP current decay in nontreated control cells did not show significant difference between two depolarization steps with 2 min interval in our condition (paired *t* test, *p* = 0.18, *n* = 10, Fig. 4*B*). In contrast, Kv7.2 + Ci-VSP current showed smaller current decay after a 2 min treatment with 10 μm DHEAS (*t* = 2 min) compared with that before DHEAS treatment (*t* = 0, paired *t* test, *p* = 0.028, *n* = 12, Fig. 4*C*). To confirm protection of Kv7 current from PIP2 depletion by DHEAS, we also tested another version of VSP, eVSP, which is an improved version of Dr-VSP and requires stronger depolarization for enzyme activation. Effects of PIP2 depletion in this condition were tested as follows: first, control Kv7.2 current was elicited by a step depolarization to −20 mV for 2 s, then depleted PIP2 by activating eVSP at 100 mV for 1 s, followed by at a test step to −20 mV for 4 s to observe remaining Kv7.2 currents (Fig. 4*D*,*E*). Because of contamination of deactivating tail currents after 100 mV steps, we compared current amplitudes at 1.5 s of −20 mV test pulse shown in Figure 4*E*. Obtained results were consistent with those obtained from Ci-VSP, and 10 μm DHEAS increased residual Kv7.2 current after PIP2 depletion (paired *t* test, *p* = 0.009, *n* = 8, Fig. 4*F*). These results indicate that DHEAS-treated Kv7.2 currents became more resistant to PIP2 depletion.

### The binding site of DHEAS in Kv7 subunit is located at the cytosolic tail surrounding histidine residue 559

**Figure 5.**
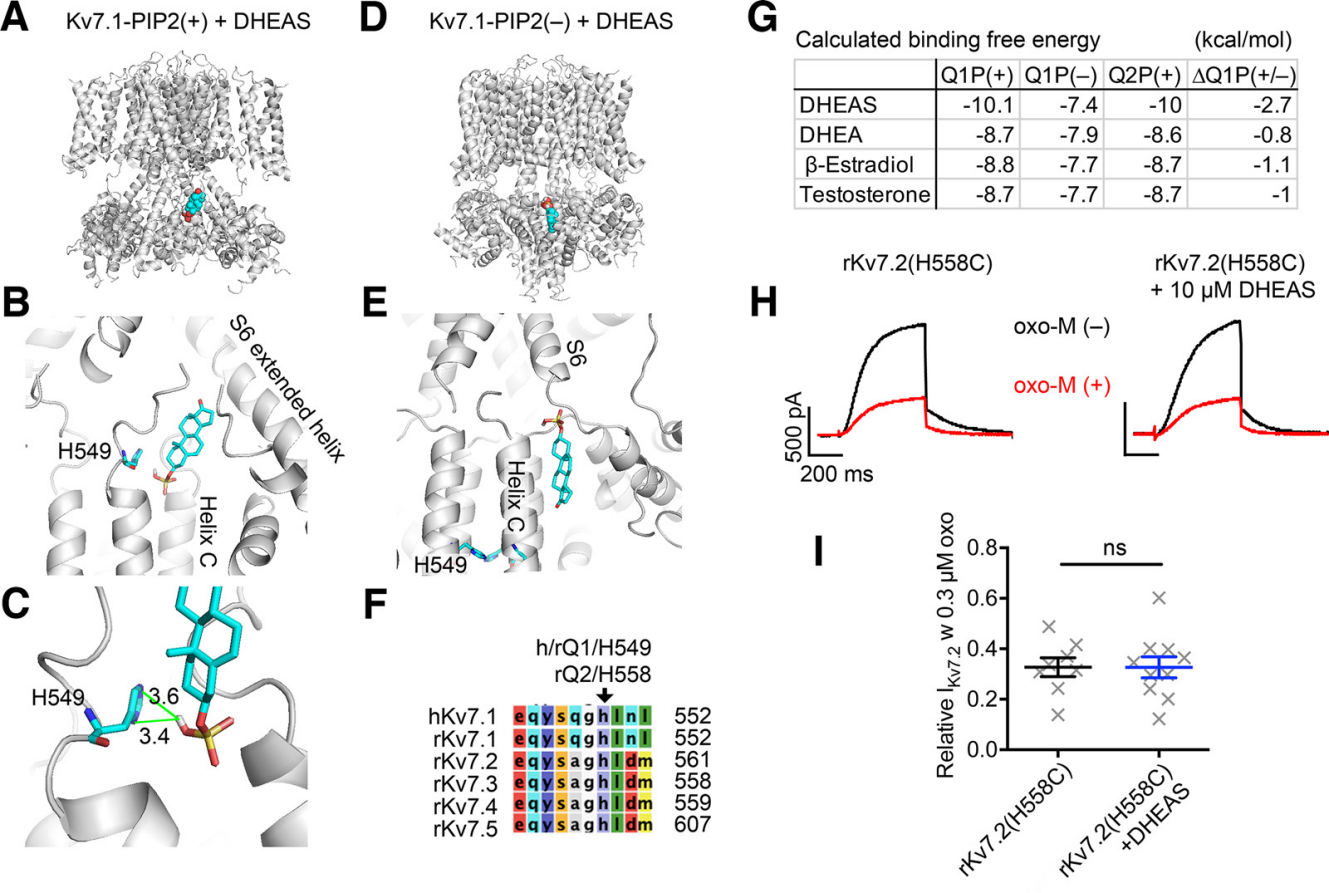
Computer modeling of DHEAS binding to Kv7 channel complex. ***A***, Whole-channel view of an ion-conducting conformation of the Kv7.1 complex in the presence of PIP2 [Kv7.1-PIP2(+)] in a gray ribbon diagram with bound DHEAS in a space-filling model [carbon (cyan), oxygen (red), sulfur (yellow)] at the cytosolic domain. ***B***, Enlarged image of a ribbon diagram of Kv7.1 and bound DHEAS in a stick model. Names of surrounding signature domains of the Kv7.1 subunit, and a candidate interacting histidine residue (H549) are indicated. ***C***, Expanded view of DHEAS and H549. Numbers indicate distances in angstrom (A°). ***D***, Whole-channel view of an inactive conformation of Kv7.1 complex without PIP2 [Kv7.1-PIP2(–)] in a gray ribbon diagram with bound DHEAS in a space-filling model. ***E***, Enlarged image of Kv7.1-PIP2(–) and bound DHEAS in stick model. Surrounding signature domains of the Kv7.1 subunit and candidate interacting histidine residue (H549) are indicated. ***F***, Amino acid sequence alignment of Kv7 channel family surrounding the candidate interacting histidine. Residue numbers of human Kv7.1 and rat Kv7.1-7.5 are indicated. Arrow indicates the candidate interacting histidine and its residue numbers in h/rKv7.1 and in rKv7.2 subunits. ***G***, Table summarizing calculated binding free energy of indicated steroid hormones to Kv7.1-PIP2(+) [Q1P(+)], Kv7.1-PIP2(–) [Q1P(–)], Kv7.2-PIP2(+) [Q2P(+)], and energy gaps between Kv7.1-PIP(+) vs Kv7.1-PIP2(–) [ΔQ1P(+/–)]. ***H***, Representative current traces of rat Kv7.2(H558C) mutant channels and its response to 1 min application of 0.3 μm oxo-M with or without 10 μm DHEAS pretreatment. ***I***, Scatter plot summarizing individual responses to 1 min application of 0.3 μm oxo-M. Data are mean ± SEM. Not significant > *p* = 0.05.

Our results thus far suggest direct action of DHEAS on Kv7 subunits. To identify the binding site of DHEAS in Kv7 channels, we conducted *in silico* binding analysis using Autodock vina software (Trott and Olson, 2010). Since the Kv7 channel family has a highly conserved modular structure, we used the published structure of the ion-conducting conformation of K7 channels from the Kv7.1-KCNE3-CaM channel complex in the presence of PIP2 (Sun and MacKinnon, 2020). To eliminate effects from KCNE3 in our analyses, KCNE3 was deleted from the published structure for our template, the structure designated as Kv7.1-PIP2(+) hereafter. To evaluate binding, we conducted a series of binding simulations for DHEAS against various subdomains in the Kv7.1-PIP2(+) defined by grid boxes. A series of binding simulations identified a high-affinity binding site in the Kv7.1-PIP2(+) with a binding free energy of −10.1 kcal/mol at a cytosolic intramolecular cavity surrounded by the S6 extended helix and the helix C (Fig. 5*A*,*B*,*G*). To estimate interaction of DHEAS with Kv7.2 subunits, we computationally replaced amino-acid residues surrounding the predicted binding pocket of Kv7.1-PIP2(+) to those of Kv7.2 subunit, Q2P(+). The predicted binding free energies were similar between Kv7.1 and Q2P(+) (Fig. 5*G*), suggesting a conserved mechanism for the interaction. Indeed, our binding model indicated a potential interaction between the sulfate moiety of DHEAS and the histidine residue (H549) in human Kv7.1 (Fig. 5*C*), which is conserved among Kv7.1-7.5 subtypes (Fig. 5*F*). We also conducted a similar analysis on the inactive conformation of Kv7.1-CaM structure without PIP2, designated as Kv7.1-PIP2(–), derived from the published structure (Sun and MacKinnon, 2020). A DHEAS binding model for Kv7.1-PIP2(–) showed a less negative binding free energy with a distinct binding configuration of DHEAS in relation with the Kv7.1 subunit with a loss of interaction with H549 (Fig. 5*E*,*G*). We also performed binding simulations with related steroid hormones that we tested in our patch-clamp experiments. All steroid hormones were predicted to bind at the same intramolecular pocket with similar configurations. Interestingly, only DHEAS showed clear difference in binding free energy between two conformations [PIP(+) vs PIP2(–)] (Fig. 5*G*), which may explain why only DHEAS can stabilize the PIP(+) conformation. After these analyses, a Kv7.4 channel structure in the presence of PIP2 was published (Zheng et al., 2022). To our surprise, similar binding analysis gave a weaker binding free energy (−7 kcal/mol) between DHEAS and Kv7.4-PIP2(+), which was not consistent with our electrophysiological results. Close inspection of the Kv7.4 structure revealed that the peptide loop preceding the helix C intersects the predicted binding space, while the loop in the Kv7.1 structure appeared to be unstructured; this distinction may suggest flexibility of this region. Because Autodock vina treats protein templates as rigid, inflexible structures (Trott and Olson, 2010), flexibility of this domain may contribute to this discrepancy. Since Kv7.4-DHEAS binding simulation did not support our results from electrophysiological experiments, we did not further analyze the Kv7.4 structure in this study.

Since our primary interest was to identify the binding site within Kv7 subunits, next we tested whether the candidate interacting histidine residue, shown in Figure 5*C*, indeed plays a role in DHEAS binding in the Kv7.2 subunit. The equivalent histidine position of human Kv7.1(H549) in rat Kv7.2 is H558. We made a cysteine substitution mutation, rat Kv7.2(H558C), to examine the contribution of this histidine residue (Fig. 5*G*). When current suppression was tested with 0.3 μm oxo-M, rKv7.2(H558C) current showed equivalent current suppression to that of WT rKv7.2 current (0.28 ± 0.4 of the reference amplitudes, *n* = 8). Importantly, DHEAS lost its effects and showed a similar degree of current suppression (DHEAS, 0.32 ± 0.05 of reference amplitudes, *n* = 10, *p* = 0.76, Mann–Whitney test, Fig. 5*I*,*J*). These results suggest that an intramolecular space surrounding the histidine residue [rat Kv7.2(H558)] is the binding site for DHEAS.

### DHEAS administration reduced inflammatory pain

**Figure 6.**
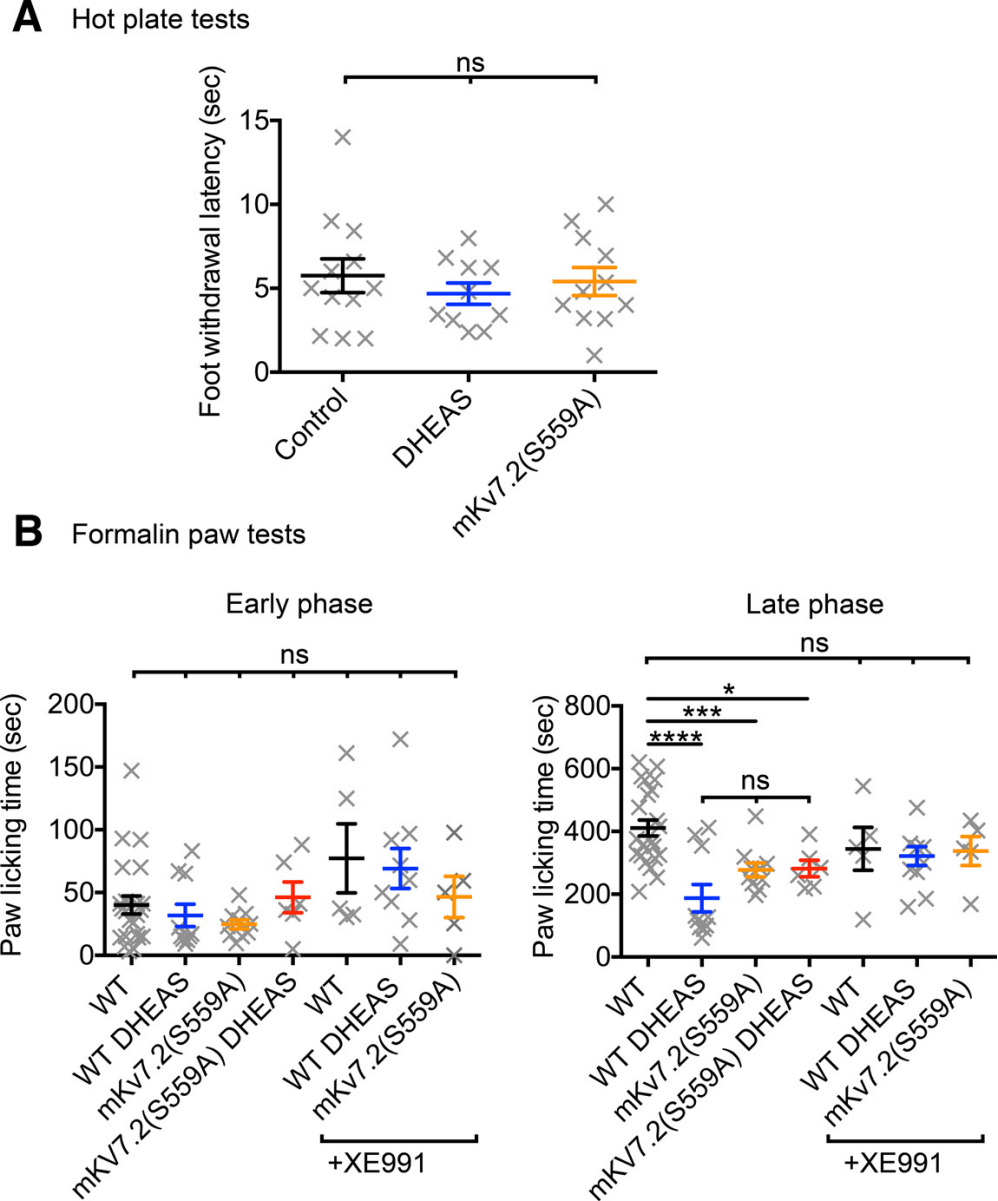
Effects of DHEAS on rodent pain models. ***A***, Summary of hot plate test. Each symbol represents an individual mouse. ***B***, Summary of early phase and late phase responses of formalin paw test. Each symbol represents an individual mouse. Results from vehicle control (WT), WT + DHEAS, Kv7.2(S559A), and Kv7.2(S559A) + DHEAS mice are shown. Underlined group indicates XE991(2 mg/kg) coinjected mice. Data are mean ± SEM. Not significant > *p* = 0.05. * < *p* = 0.05. *** < *p* = 0.001. **** < *p* = 0.0001.

We next examined whether DHEAS can modify *in vivo* responses mediated by M-current suppression and related hyperexcitation of neurons. Our previous studies demonstrated that the mutant Kv7.2 channels [rat Kv7.2(S541A) (Fig. 2*D*)/mouse Kv7.2(S559A)], which lack the essential PKC acceptor site, can reduce the suppression of the M-current (Hoshi et al., 2003) and exhibit both antiseizure and neuroprotective effects in the brain (Greene et al., 2018). On the other hand, circulating DHEAS does not cross the blood–brain barrier because DHEAS is a charged molecule. In addition, DHEAS in the brain is synthesized locally from DHEA, which makes controlling DHEAS levels in the CNS extremely difficult (Maninger et al., 2009); thus, we focused on other effects. Since flupirtine and retigabine show analgesic effects via Kv7 channel mechanisms involving dorsal root ganglion neurons (Passmore et al., 2003), we tested effects of DHEAS on pain. To determine effects on DHEAS on basal and sensitized nociception, we conducted hot plate tests and formalin paw tests in WT mice. We also tested Kv7.2(S559A) knock-in mice, which have attenuated M-current suppression derived from the equivalent mutation to rat Kv7.2(S541A) (Fig. 2*D*). Results of the hot plate test, which evaluates thermal nociception, showed no differences among control, DHEA-treated WT mice, and Kv7.2(S559A) mice (*p* = 0.8, Kruskal–Wallis test), which suggest intact basal nociception (Fig. 6*A*). Next, we conducted the formalin paw test. The early phase responses of formalin tests from control, DHEA-treated WT mice, and Kv7.2(S559A) mice did not show significant differences (Fig. 6*B*). On the other hand, the late phase showed reduced pain responses in DHEAS-treated WT as well as in Kv7.2(S559A) mice with or without DHEAS (Fig. 6*B*; two-way ANOVA; interaction: *F*_(6,120)_ = 4.50, *p* = 0.0004; phases: *F*_(1,120)_ = 258.0, *p* < 0.0001, treatments: *F*_(6,120)_ = 5.48, *p* < 0.0001). Administration of XE991 abolished analgesic effects or phenotypes of DHEAS and Kv7.2(S559A) knock-in mice, which suggest that Kv7 channel activities are responsible for these effects (Fig. 6*B*). The early phase of formalin paw tests detect chemical activation of acute nociception while the late phase of formalin paw test detect nociception sensitized by inflammatory mediators released from damaged tissues (Bannon and Malmberg, 2007). These results suggest that DHEAS treatment as well as mKv7.2(S559A) mutation mitigate sensitized inflammatory pain through Kv7 channel-mediated mechanisms.

## Discussion

This study demonstrates that DHEAS is an endogenous Kv7 channel modulator that attenuates M-current suppression. This is a novel type of Kv7 channel modulation that reduces responsiveness of M-current suppression to Gq-coupled receptor stimulation. This novel type of Kv7 channel modulation is distinct from the effects by existing first-generation Kv7 modulators, such as retigabine, in that it does not change voltage-dependent kinetics or basal activities of Kv7 channels. We determined the binding site of DHEAS in Kv7 subunits at a cytosolic intramolecular cavity surrounding by the S6 extended helix and the helix C domain, which has never been considered as a binding site for small molecules. We have also demonstrated that DHEAS administration can reduce inflammatory pain in rodent. Because XE991 abolished analgesic effects of DHEAS and mKv7.2(S559A) mutation, the analgesic effects in these conditions were likely mediated by preserved Kv7 channel activity during inflammation as a result of reduced M-current suppression. This study demonstrated a novel regulation of Kv7 channels, elucidating the dynamic control of M-current suppression sensitivity by small molecules in both physiological and pathologic conditions. We believe that the identification of this regulatory pathway holds significant implications for our understanding of channel function and provides potential insights for regulation of neuronal excitability. Namely, this mechanism can provide fine-tuning of regulation on neuronal excitability by hormonal factors. We propose that molecules that mimic DHEAS can be used to suppress pathologic neuronal hyperexcitation.

One noticeable finding in this study is the immediate onset of DHEAS effects, which occurs within 2 min *in vitro* (Fig. 4*B*) and 30 min *in vivo* (Fig. 6). This fast onset of DHEAS effects makes a clear contrast to the slow responses of nuclear receptor pathways mediated by steroid hormone receptors, which suggests a distinct mechanism. Our results shown in Figure 3 suggest that DHEAS is unlikely to modulate m1 muscarinic receptor signaling per se because oxo-M-induced PIP2 metabolism and activation of PKC remained unaltered after DHEAS treatment. On the other hand, direct effects of DHEA/DHEAS on ion channels have been reported for GABA_A_ and NMDA ionotropic receptors (Majewska and Chuang, 1984; Bergeron et al., 1996; Maninger et al., 2009). As shown in Figure 5, our computational binding analyses and observed loss of DHEA effects by rKv7.2(H558C) mutation support the direct binding and direct action of DHEAS on Kv7 channels.

Regarding a mechanism for attenuating muscarinic suppression of Kv7 currents, our results from Ci-VSP/eVSP-mediated PIP2 depletion experiments indicates that DHEAS confers resistance to Kv7 channels to decreased PIP2 levels, suggesting stabilization of Kv7-PIP2 interaction.

According to our computational models, the binding of DHEAS to Kv7.1-PIP2(+) has a lower binding free energy of −2.7 kcal/mol compared with that of DHEAS and Kv7.1-PIP2(–) (Fig. 5*H*). This energy gap created by DHEAS binding may function as an additional energy barrier for conformational transitions stabilizing the PIP(+) conformation. Surprisingly, the reported structure of the Kv7.4 channel did not show a high-affinity binding of DHEAS in our computational models. As discussed earlier, one possibility is because of the limitation of the computer program to incorporate the flexibility of protein structures. Another possibility is that the Kv7.4 structure published in the literature represents an extreme condition (PIP2-saturated conformation) because this particular structure contains two PIP2 molecules per subunit (Zheng et al., 2022). Overproduction of PIP2 in cells by coexpression of phosphatidylinositol 4-phosphate 5-kinase has been known to change not only current density but also voltage dependence of Kv7 channels (K. S. Kim et al., 2016; Pant et al., 2021), including Kv7.4 channels (Lee et al., 2021). This change in channel kinetics induced by PIP2 overproduction suggests that the PIP2 binding sites of Kv7 subunits in the physiological condition are not saturated. Therefore, PIP2-unsaturated Kv7.4 channel may take a distinct conformation allowing to accept DHEAS molecules.

DHEAS is the most abundant circulating steroid hormone that reaches its maximal concentration of ∼10 μm during the ages of 20-30 in human and gradually declines with age (Ebeling and Koivisto, 1994; Baulieu and Robel, 1998). This concentration of ∼10 μm falls within the range of our experimental condition. However, serum DHEAS is known to bind strongly to albumin (Ebeling and Koivisto, 1994) and the interstitial fluid contains lower albumin. Thus, the concentration of free DHEAS surrounding tissues (including DRG neurons) should be lower than total serum DHEAS. On the other hand, whether DHEAS is concentrated at the plasma membrane as a steroid derivative or how it is transferred to Kv7 subunits remains unknown. Since this study is the first report relating DHEAS with Kv7 channels, we confined our experiments to short-term treatment for initial characterization purposes. It is possible that intracellular DHEAS did not reach equilibrium in our conditions.

It has been reported that serum DHEAS concentration drops to <20% of the peak concentration by the age of 70 (Ebeling and Koivisto, 1994). Therefore, age-dependent reduction of serum concentration of DHEAS could have a significant impact on Kv7 modulation. It has been shown that low DHEAS correlates with chronic pain in aged females in a population study (Li et al., 2021). In addition, it is possible that DHEAS affects blood pressure regulation since Kv7 channels are expressed in the vascular smooth muscle. However, several population studies reported mixed results for correlation between DHEA/DHEAS and cardiovascular diseases, including blood pressure (Schunkert et al., 1999; Carroll et al., 2011; Savineau et al., 2013). Nonetheless, our study suggests potential contribution of Kv7-mediated mechanisms to age and DHEAS-related symptoms.

A reasonable question would be whether this new type of Kv7 modulation holds potential for treating diseases. However, administering DHEAS directly to patients may not be an optimal strategy, as it is easily metabolized into other steroid hormones. Development of new small molecular weight compounds that can mimic the effects of DHEAS offers a more promising approach for medical intervention to terminate pathologic overexcitation of neurons.

In conclusion, we have identified a novel class of Kv7 channel modulators, which binds to a novel binding site in the cytoplasmic tail of Kv7 channels and attenuates M-current suppression. This novel type of Kv7 channel modulation has a potential for reducing sensitized neuronal activities, making it a promising candidate for medical intervention.
